# Supplementary material for: Using multi-label ensemble CNN classifiers to mitigate labelling inconsistencies in patch-level Gleason grading
Source: PLoS One. 2024 Jul 5;19(7):e0304847. doi: 10.1371/journal.pone.0304847 (PMC11226137; doi:10.1371/journal.pone.0304847)
Supplement: Attachment — Submitted filename: Reviewer Comments.pdf [file pone.0304847.s002.pdf]

Reviewer Comments on the article entitled.

"Using Multi-Label Ensemble CNN Classifiers to Mitigate Labelling Inconsistencies in Patch-level Gleason Grading."

- The abstract provides a clear overview of the problem addressed, the methodology employed, and the key findings, setting the stage for the reader's understanding and accurately reflects the focus of the research on addressing labeling inconsistencies in Gleason grading using multi-label ensemble CNN classifiers.
- The introduction was clear and relevant to the context of the research problem and the author provided a sufficient background on the significance of the problem. The background includes key studies and methodologies in the effectiveness of patch-level Gleason Grading of prostate histopathology images demonstrating a deep understanding of the existing literature.
- The methods section has a detailed description of the deep learning model architecture and training process. Although, some information in the results is recommended to be added to the methods instead and present the validated data in the results section. Also, the descriptive flowchart may include more information on the stated method to give a clearer image of the proposed process. Some figures require double-checking of the captions and resolution to improve presented information.
- The data used in the paper is explained and referenced to be available in future work as well as the evaluation metrics, and validation procedures. The results presentation was clear through tables, figures, and performance metrics to reflect the effectiveness of the multi-label ensemble CNN classifiers. An expansion of limitations and future directions to guide further research in the field is recommended.
- The references support the background, methodology, and findings of the research with double-checking the accuracy of references, including author names, publication years, journal/conference titles, and page numbers, to ensure credibility and readability are recommended.
- Moreover, refer to the reviewed article file for comments attached in the uploaded file.
